# AST-487 Inhibits RET Kinase Driven TERT Expression in Bladder Cancer

**DOI:** 10.3390/ijms231810819

**Published:** 2022-09-16

**Authors:** Neeraj Agarwal, Qiong Zhou, Deepak Arya, Sébastien Rinaldetti, Jason Duex, Daniel V. LaBarbera, Dan Theodorescu

**Affiliations:** 1 Cedars-Sinai Samuel Oschin Comprehensive Cancer Institute, Los Angeles, CA 90048, USA; 2 Department of Pharmaceutical Sciences, Skaggs School of Pharmacy and Pharmaceutical Sciences, Aurora, CO 80045, USA; 3 The CU Anschutz Center for Drug Discovery, The University of Colorado Anschutz Medical Campus, Aurora, CO 80045, USA; 4 The University of Colorado Cancer Center, The University of Colorado Anschutz Medical Campus, Aurora, CO 80045, USA; 5 Department of Surgery (Urology), Cedars-Sinai Medical Center, Los Angeles, CA 90048, USA; 6 Department of Pathology and Laboratory Medicine, Cedars-Sinai Medical Center, Los Angeles, CA 90048, USA

**Keywords:** TERT, telomerase, RET

## Abstract

Mutations in the promoter of the human Telomerase Reverse Transcriptase (hTERT) gene are common and associated with its elevated expression in bladder cancer, melanoma, and glioblastoma. Though these mutations and TERT overexpression are associated with aggressive disease and poor outcome, an incomplete understanding of mutant TERT regulation limits treatment options directed at this gene. Herein, we unravel a signaling pathway that leads to upregulated hTERT expression resulting from the −124 bp promoter mutation, the most frequent variant across human cancer. We employed engineered bladder cancer cells that harbor a GFP insertion at the TSS region on −124 hTERT promoter for high-content screening drug discovery using a focused library of ~800 kinase inhibitors. Studies using in vitro and in vivo models prioritized AST-487, an inhibitor of the wild-type, and mutant RET (rearranged during transfection) proto-oncogene as a novel drug inhibitor of both wild-type and mutant promoter-driven hTERT expression. We also identified the RET kinase pathway, targeted by AST-487, as a novel regulator of mutant hTERT promoter-driven transcription in bladder cancer cells. Collectively, our work provides new potential precision medicine approaches for cancer patients with upregulated hTERT expression, perhaps, especially those harboring mutations in both the RET gene and the hTERT promoter, such as in thyroid cancer.

## 1. Introduction

Human telomerase reverse transcriptase (hTERT) is commonly expressed in cells that divide rapidly, such as stem cells and cells functioning in development [1,2,3]. While hTERT expression is repressed in most normal somatic tissues, many types of cancer have developed mutations in the gene promoter region to upregulate gene expression [4,5,6]. Increased hTERT activity is important in cancer cell survival since hTERT also has non-canonical/non-telomerase roles in promoting cell proliferation, DNA-damage response, and protecting cancer cells from apoptosis [7,8,9]. Patients harboring hTERT promoter mutations have shorter survival than patients with no mutations [10]. Recurrent hTERT promoter mutations, at −124 bp and −146 bp from the translation start site (TSS), are the most frequent events associated with hTERT promoter alterations and mRNA overexpression in cancer [10]. Importantly, these mutations are heterozygous and mutually exclusive in mutant cancer cell lines [5]. Furthermore, an identical DNA sequence created by both 124 and 146 mutations leads to the formation of de novo binding sites for ETS (E-twenty-six) transcriptions factors [11], including GA Binding Protein Transcription Factor Alpha (GABPA) [12,13]. Strikingly, hTERT promoter mutations are detected at high frequency in the bladder (70%), glioma (67%), thyroid (60%), and melanoma (49%) cancers [10]. Although the impact of hTERT promoter mutations has been characterized in human cancer, the signaling pathways upstream of the transcription factors above, and the means to inhibit those pathways, remain relatively unexplored.

In this study, we set out to identify those upstream signaling pathways that drive hTERT transcription from the mutant promoter at −124. By employing a novel reporter in bladder cancer (BLCA) cells and a kinase inhibitor library, we also aimed to identify kinase inhibitors of hTERT upregulation. We previously generated a novel engineered reporter cell line (UMG12) by knocking-in green fluorescent protein (GFP) at mutant (−124) hTERT promoter allele in UMUC3 human BLCA cells [14]. Using the UMG12 cell model system, we performed high-content screening (HCS) with ~800 commercially available kinase inhibitors. HCS identified 21 hits with no previous association in altering hTERT expression. Hit-to-Lead compound validation studies using monolayer and tumor organoid cell models, and in vivo xenograft tumors, prioritized AST-487 as a lead compound that inhibits mutant promoter-driven hTERT expression by inhibiting upstream RET kinase receptor activity. Genomic depletion confirmed the role of RET in hTERT expression. Collectively, this work links RET kinase as a key regulator of mutant promoter-driven hTERT expression and proposes a novel potential precision medicine strategy for cancer patients harboring mutant hTERT promoter sites. This approach may be particularly suitable for patients with a high frequency of RET and TERT alterations, such as those with thyroid cancer [10,15,16,17,18].

## 2. Results

### 2.1. Drug Discovery Targeting Mutant Promoter Driven hTERT Expression

**Figure 1:**
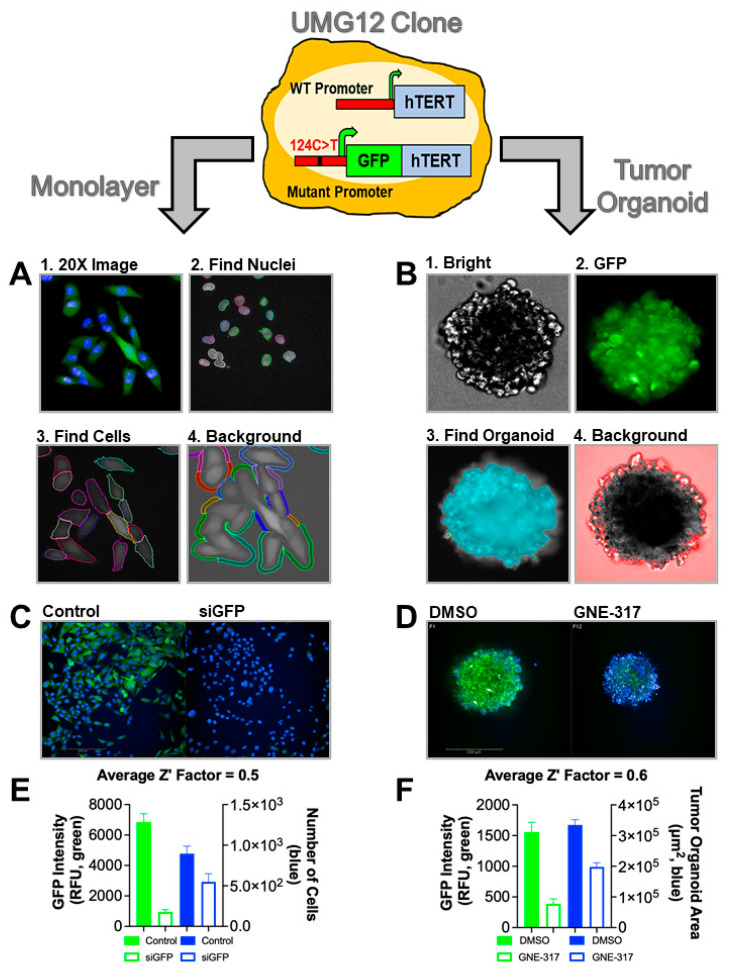
**HCS assay validation in UMG12 cell models.** UMUC3 BLCA cells engineered with a mutant promoter-driven GFP-hTERT fusion reporter cell line (UMG12) were developed and validated for HCS drug discovery in cells cultured as monolayers or tumor organoids. (**A**) monolayer and (**B**) tumor organoid Step-wise high-content analysis workflow of images acquired after HCS. (**C**) Monolayer and (**D**) Tumor organoid representative images of HCS controls showing GFP intensity after the PI3K inhibitor GNE-317 (1 μM, +control), a known inhibitor of hTERT expression. (**E**) Monolayer and (**F**) Tumor organoid Z’-factor analysis demonstrating valid controls for HCS.

UMG12 GFP-hTERT fusion reporter cells were used to generate monolayer and tumor organoid models for High-Content Screening (HCS) drug discovery of a focused library of ~800 kinase inhibitors (Figure 1). Each cell model was treated with kinase inhibitors for 72 h before high-content imaging on an Opera Phenix HCS System. Images were analyzed with Harmony High-Content Imaging and Analysis Software version 4.9 (PerkinElmer, Waltham, MA, USA). Mean GFP intensity was calculated with a 4-step method (Figure 1A,B). For monolayer imaging, images were acquired, nuclei were identified by Hoechst 33342 staining (Thermo Scientific, Waltham, MA, USA), cell boundaries were then determined by measuring cytoplasmic GFP fluorescence and brightfield imaging, and finally, the background signal around each cell was measured and subtracted from the cell GFP signal. Mean GFP intensity from all cells in random fields within a single well was then calculated. Tumor organoid images were captured by measuring brightfield, Hoechst 33343, and GFP channels, and the organoid area was measured using brightfield images. To measure GFP intensity from each tumor organoid, the background signal around the tumor organoid was subtracted from the GFP signal of each tumor organoid. Assay conditions were standardized using mock siRNA control (−control), GFP-siRNA treatment (+control), DMSO (-control), or GNE-317, a PI3K inhibitor and positive control known to downregulate hTERT expression (Figure 1C,D) [19,20]. The Z’-factor analysis for each plate was employed for GFP intensity and cell viability, validating both cell models for HCS (Figure 1E,F) [21,22].

**Figure 2:**
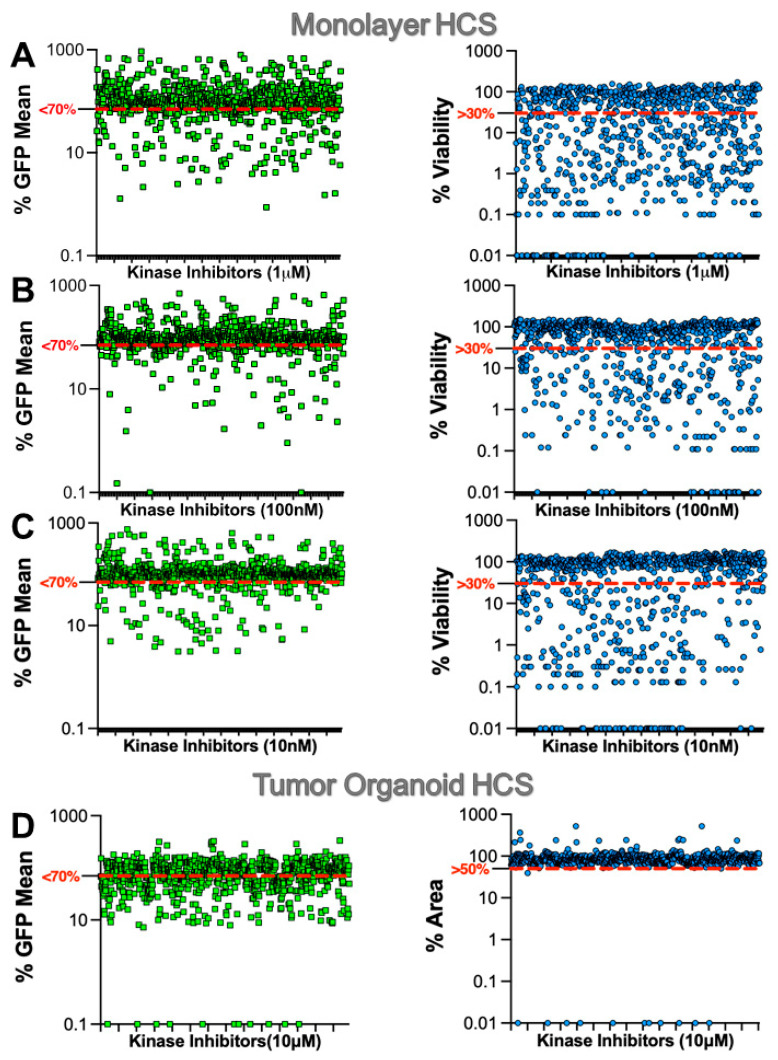
**HCS of kinase inhibitors in UMG12 cell models.** UMG12s were used to screen a focused library of kinase inhibitors in cells cultured as (**A**–**C**) monolayers or (**D**) tumor organoids. HCS of monolayer cells were treated with 3 different concentrations of kinase inhibitor library as indicated. The hit limit for mean GFP intensity was set to less than 70% (red dashed line) for each HCS condition. In addition, cell viability was multiplexed with GFP mean intensity with a hit limit set at great than 30% (monolayer) and 50% (tumor organoid) viability, respectively. Both GFP and viability hit limits were used to select hits from HCS.

Next, the UMG12 cells were used in a monolayer to conduct focused HCS with kinase inhibitors arrayed with three log doses of 10 nM, 100 nM, and 1 µM. Tumor organoids proved to be less sensitive to kinase inhibitors than monolayer culture, and therefore, organoids were screened at a single dose of 10 µM (Figure 2). For both monolayer and tumor organoid HCS, most of the kinase inhibitors had little or no effect on GFP-hTERT expression (%GFP mean) at all concentrations tested with a hit limit set at <70%. Because kinase inhibitors are well known to alter tumor cell growth or viability, we multiplexed cell viability in conjunction with GFP-hTERT expression by measuring the % viability. These viability values were based on differences in cell numbers between DMSO and kinase inhibitor-treated monolayer cells or by measuring the percentage area of tumor organoids.

**Figure 3:**
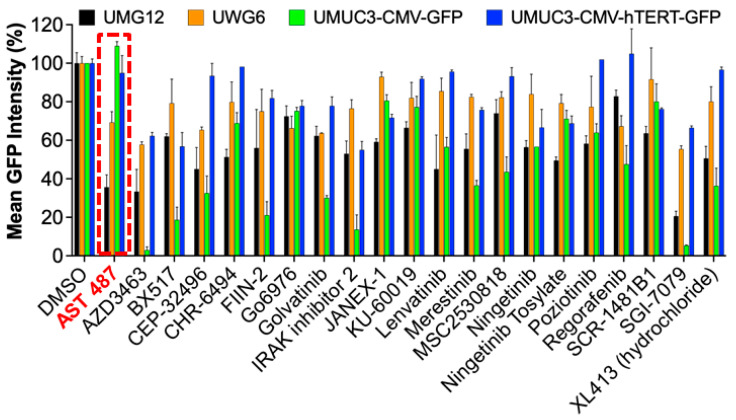
**HCS identified 21 hits targeting 16 kinases with no known association to hTERT.** The Mean GFP intensity was measured in UMG12, UWG6, UMUC3-CMV-GFP, and UMUC3-CMV-hTERT-GFP cells treated with 21 kinase inhibitors. Red dotted line box indicate the effect of AST-487 on cell proliferation.

To select and prioritize hits from HCS, we first excluded hits that lead to <30% in cell viability (monolayer) and <50% area (tumor organoid) to deprioritize hits that significantly alter cell viability. The reasons for excluding compounds that suppress cell viability when screening for compounds that inhibit transcription of TERT were as follows: (1) reliability and robustness of transcriptional readouts are compromised in cells whose viability is severely compromised by drugs; (2) the specificity of the reduced TERT expression readout may be compromised by the simultaneous activity of cell death machinery; (3) we sought to identify relatively specific agents that rapidly reduced TERT expression rather than those which also rapidly inhibited growth since the latter would not be due to TERT depletion. Compounds that passed these criteria and decreased mean GFP intensity by <70% were considered hits on both screens. These hit criteria and filtering identified 125 hits from monolayer HCS and 249 from the tumor organoid HCS. Of these, 78 hits were identified from both screens hitting 25 different known kinases (Table S2). Next, we performed a literature analysis to determine how many of these 25 kinases have been reported to regulate hTERT expression. We found that PI3K [19], EGFR [23], mTOR [14], AKT [19], Raf [24], MEK [23,24], BCR-ABL [25], p38MAPK [26], and ERK [24] are already reported to affect hTERT expression. Hence, we excluded 57 inhibitors targeting these kinases from further studies, as our goal was to identify only novel kinase inhibitors that downregulate hTERT expression. This filtering reduced the hits of interest to 21, targeting 16 kinases. The 21 hits were confirmed by rescreening in 3D at 10 μM concentration (Figure 3). In addition, we also confirmed hits using three other orthogonal cell line models, including the UWG6 clonal cell line [14], in which GFP is tagged to the wild-type hTERT promoter, UMUC3-CMV-GFP with CMV-promoter driven exogenous GFP expression, and UMUC3-CMV-hTERT-GFP with CMV-promoter driven exogenous hTERT-GFP expression. From these hit confirmation studies, the kinase inhibitor AST-487 displayed the most promising activity as it significantly inhibited GFP-hTERT expression from the mutant hTERT promoter in UMG12 cells and from the wild-type hTERT promoter in UWG6 cells. In contrast, AST-487 did not inhibit the CMV promoter-driven GFP or hTERT-GFP expression. Other inhibitors either did not significantly inhibit the hTERT promoter-driven GFP-hTERT expression or inhibited the CMV-driven GFP expression.

To confirm hits from HCS, we utilized four orthogonal cell lines, including UMG12 (mutant promoter driving GFP-hTERT fusion protein), UWG6 (wt promoter driving GFP-hTERT fusion protein), UMUC3-CMV-GFP (GFP control), and UMUC3-CMV-hTERT-GFP (hTERT-GFP fusion protein control) as indicated. Taken together, hits were confirmed based on the downregulation of GFP intensity in UMG12 and UWG6 but not the control cell models. Based on these criteria, AST487 (red dashed box) was prioritized for hit-to-lead validation studies.

### 2.2. AST-487 Inhibits hTERT Expression In Vitro and In Vivo

**Figure 4:**
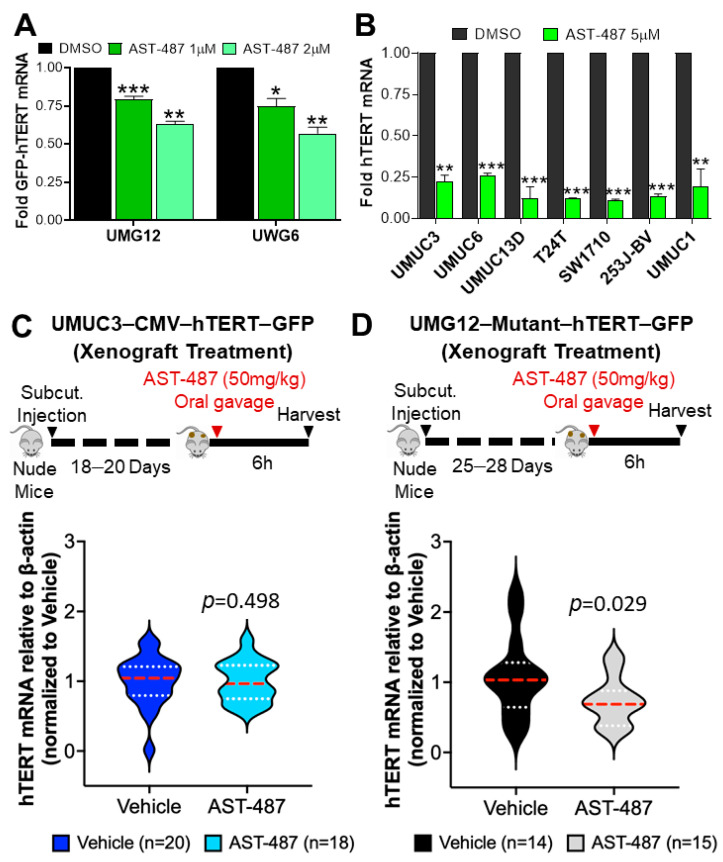
**Validation of AST-487 for effect on hTERT expression in vitro and in vivo.** (**A**) GFP-hTERT mRNA expression from UMG12 and UWG6 cells were treated with AST-487, as indicated, for 16 h, followed by qRT-PCR analysis. (**B**) hTERT mRNA expression from different BLCA cells was treated with AST-487 for 16 h, followed by qRT-PCR analysis. Schematics showing (**C**) UMUC3-CMV-hTERT-GFP and (**D**) UMG12 tumor xenografts development and treatment with AST-487 in nude mice as indicated on the top of the graph panels. Tumors were harvested, followed by qRT-PCR analysis. hTERT mRNA expression is shown (bottom of the graph panels) from (**C**) UMUC3-CMV-hTERT-GFP and (**D**) UMG12 xenografts 6 h post AST-487 treatment. The significance is indicated as: * *p* < 0.05, ** *p* < 0.01, *** *p* < 0.001.

To further confirm that AST-487 is inhibiting hTERT expression from both the mutant and wild-type promoter and validate the results from HCS and orthogonal assays, GFP-hTERT mRNA levels were measured by qRT-PCR after AST-487 treatment in UMG12 and UWG6 cells. A reduction in GFP-hTERT mRNA levels occurred after AST-487 treatment in both UMG12 and UWG6 cells, which correlated with a decrease in GFP-reporter activity (Figure 4A and Figure S1). To examine the potential broader impact of AST-487 activity beyond engineered cell lines, we examined the effect of AST-487 on native hTERT mRNA levels in multiple human bladder cancer cell lines, treating cells at 5 μM concentration over 16 h. We observed a>75% decrease in hTERT mRNA levels in all the cell lines tested after treatment with AST-487, regardless of their hTERT promoter mutation status (Figure 4B). We next examined the ability of AST-487 to inhibit hTERT expression in vivo by employing UMG12 and UMUC3-CMV-hTERT-GFP in nude mouse tumor xenografts. The mice were administered AST-487 by oral gavage, and 6 h later tumors were harvested and hTERT expression was measured by qRT-PCR (Figure 4C,D). As expected, hTERT expression in UMUC3-CMV-hTERT-GFP control tumors was unaltered after AST-487 treatment (Figure 4C). In contrast, AST-487 treatment significantly reduced hTERT expression in UMG12 xenografts (Figure 4D). Collectively, these results support the in vitro results, demonstrating that AST-487 is an effective lead compound inhibiting hTERT promoter activity.

### 2.3. AST-487 Suppresses hTERT Expression by Inhibiting the RET Kinase Receptor

**Figure 5:**
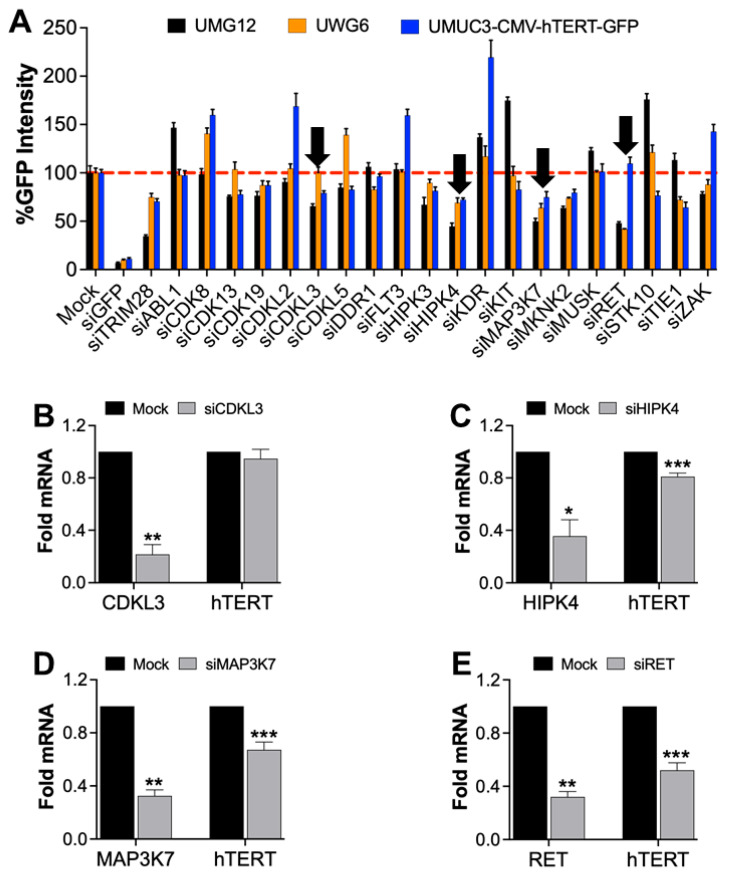
**Kinase target validation for AST-487 affecting hTERT expression.** (**A**) AST-487 inhibits several kinases, which were transiently knocked down in cells as indicated and monitored for GFP intensity after 72 h. Kinases affecting hTERT transcription are shown with black arrows. (**B**–**E**) T24T BLCA cells were treated with siRNAs as indicated, and the knockdown of genes and effect on hTERT mRNA was confirmed by qRT-PCR. The student’s t-test indicated significance: * *p* < 0.05, ** *p* < 0.01, *** *p* < 0.001.

AST-487 inhibits multiple kinases such as Flt3, RET, CDKL3/5, HIPK4, and MAP3K7 [27,28,29]. To deconvolute the kinase molecular target of AST-487 involved in regulating hTERT expression, we first considered a panel of kinases to which AST-487 displays potent binding affinity (Kd < 10 nM) [29] and targeted these kinases using Smartpool siRNA transfections of UMG12, UWG6, and UMUC3-CMV-hTERT-GFP cells (Figure 5A). GFP intensity was measured 72 h after siRNA transfection, and TRIM28 [14] and GFP siRNA were used as positive controls for this experiment. Notably, siRNAs against CDKL3, HIPK4, and MAP3K7 reduced GFP intensity relative to control in UMG12 and UWG6 cells but also in the control UMUC3-CMV-hTERT-GFP cells (Figure 5A). However, siRNA against the RET kinase receptor showed much higher inhibition of GFP intensity in both UMG12 and UWG6 cells but not in UMUC3-CMV-hTERT-GFP cells. These data point to RET kinase as a regulator of hTERT expression from both mutant and wild-type promoter alleles, consistent with RET kinase being an important molecular target of AST-487. Confirmation knockdown studies of CDKL3, HIPK4, MAP3K7, and RET were conducted using parental T24T cells, which harbor the heterozygous −124 promoter mutation and display higher hTERT mRNA compared to other BLCA cell lines (Figure 5B–E) [30]. These results are consistent with RET being the likely kinase target of AST-487 regulating hTERT expression in BLCA (Figure 5E).

### 2.4. AST-487 Suppresses the Proliferation of Bladder Cancer Cells

**Figure 6:**
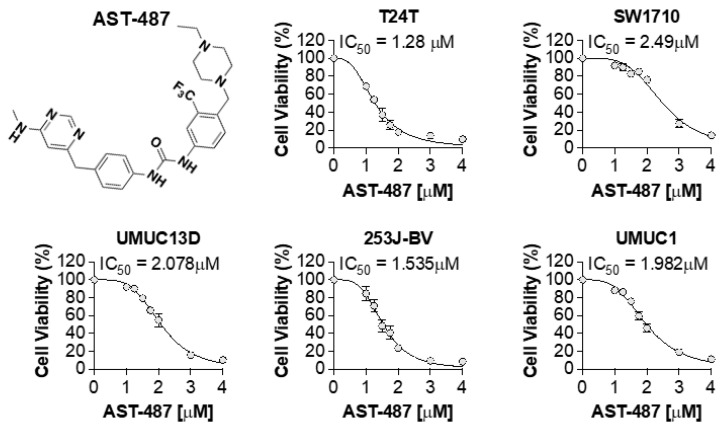
**AST-487 cell viability dose-response studies.** The chemical structure of AST-487. BLCA cell lines were treated with various concentrations of AST-487 for 72 h measuring cell viability and IC_50_ values.

In addition to inhibiting hTERT expression, we investigated whether AST-487 can also suppress BLCA cell growth. We conducted dose response studies with AST-487 using five BLCA cell lines. A dose-dependent decrease in cell proliferation was observed in all the cell lines tested (Figure 6). The inhibition concentration 50% (IC50) value for AST-487 ranged between 1.3- to 2.5-μM for all the cell lines tested. These results suggest that AST-487 treatment has therapeutic potential in treating BLCA patients with elevated hTERT expression. Surprisingly, 30–40% of BLCA don’t exhibit any mutation in the promoter region. Therefore, hTERT expression was examined in the TCGA database with no promoter region mutation. Interestingly, hTERT was overexpressed in several cancer types (Figure S2), indicating that AST-487 may be useful in treating cancers with upregulated hTERT expression even when hTERT promoters are not mutated. Finally, mutational analysis by sequencing 10,000 metastatic cancer patients [10] revealed that co-occurrence of hTERT mutations and RET mutations occur more frequently than expected when all tumors are considered (Figure S3).

## 3. Discussion

Herein, we report a comprehensive HCS campaign to identify kinase inhibitors that can effectively inhibit elevated hTERT expression, including those resulting from hTERT promoter mutations in BLCA. Our HCS approach featured both monolayer and tumor organoid cell models, which can differ significantly in gene expression and therapy response. Examining both provides a more complete picture of inhibitor effectiveness [31,32]. In addition, for each model, we multiplexed cell viability and mutant and/or wild-type promoter-driven GFP-hTERT expression to filter out pleotropic inhibitors and capture the most effective kinase inhibitors downregulating hTERT expression. We identified hundreds of hits using the HCS models, with 78 hits targeting 25 different kinases that were effective in both. Nine kinases targeted by 57 hits were eliminated from our screen because of their known effects on hTERT expression. Using our HCS approach, we discovered 21 novel inhibitors of hTERT expression, targeting 16 different kinases. Secondary orthogonal cell model screening identified AST-487 as a potential lead compound inhibitor of both mutant and wild-type promoter-driven hTERT expression.

**Figure 7:**
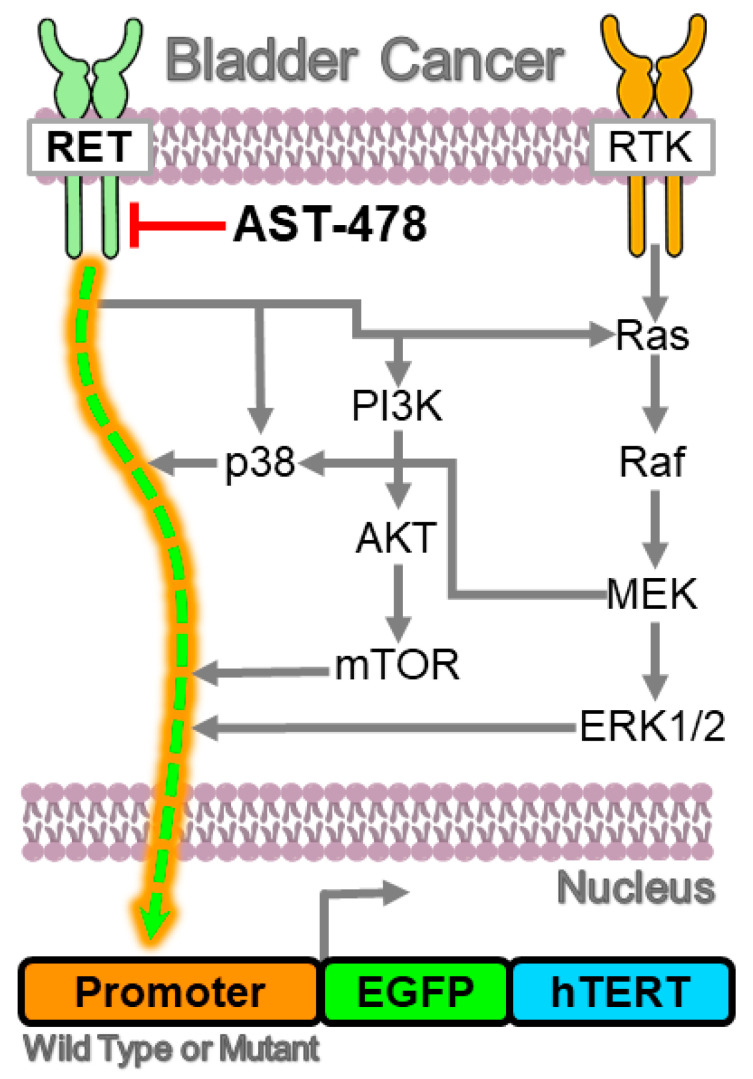
**Summary of mechanisms regulating hTERT expression in BLCA.** The results herein link the receptor tyrosine kinase RET as a key factor regulating hTERT expression in BLCA (green & orange dashed arrow). Parallel kinase signaling pathways known to regulate hTERT expression that are also activated by RET are shown with grey arrows and likely converge on upregulating hTERT expression. Upstream inhibition of RET attenuates hTERT promoter activation, including the more tumorigenic mutant hTERT promoter, regardless of the signaling pathways shown in grey.

AST-487 has been reported to inhibit a variety of receptor tyrosine kinases [29]. Therefore, there was a considerable challenge in identifying the kinase which regulates hTERT expression. However, our target deconvolution approach focused on a panel of 16 kinases with low nM binding affinities to AST-487. AST-487 is known to inhibit the autophosphorylation of RET and, consequently, downstream RET-mediated signaling cascades (Figure 7) [15,16,17,18]. In addition, the proliferation rate of thyroid cancer cells with RET kinase mutations has been shown to be inhibited by AST-487 treatment [15], which is interesting since thyroid cancer has a high rate of hTERT promoter mutations [10]. Davis et al. have profiled a total of 72 kinase inhibitors, including AST-487, which was found to be promiscuous with several different kinases [29]. We evaluated the most potent targets of AST-487 using siRNA. We found HIPK4, MAP3K7, and RET to be inhibitors of hTERT expression in T24T BLCA cells, which have an hTERT mutated promoter as well as the highest hTERT expression among all BLCA cell lines [30]. siRNA knockdown of MAP3K7 and HIPK4 also downregulated control UMUC3-CMV-hTERT-GFP cells. However, knockdown of RET did not alter CMV-driven reporter activity but had a significant impact on wt and mutant promoter-driven hTERT expression, confirming RET as a major cellular target.

RET is a transmembrane receptor kinase and upstream regulator of major oncogenic pathways such as PI3K-AKT-mTOR, Ras-ERK, JNK, and p38MAPK [33,34,35,36]. RET is activated through the glial cell line-derived neurotrophic factor (GDNF) family of ligands and has been reported to be activated in several cancer types [37]. RET signaling is constitutively activated through mutations in many cancers, notably medullary and papillary thyroid and non-small cell lung cancers [36], which also have a high rate of hTERT promoter mutations [10]. To the best of our knowledge, this is the first study reporting RET as a novel regulator of hTERT expression, including the expression driven by mutations in the hTERT promoter. RET might mediate hTERT promoter activity through its downstream signaling noted above and in Figure 7. For example, alterations in PI3K-AKT pathway genes are more common in hTERT wild-type compared to mutant hTERT promoter activity in glioblastoma and AKT phosphorylation is associated with hTERT expression in gastric cancer [38,39]. Likewise, activation of Ras MAPK signaling is also known to regulate mutant hTERT promotor activity in melanoma [24]. Recently, we demonstrated that the mTORC1 complex upregulates hTERT expression preferentially from mutant promoter through phosphorylation of the transcription factor TRIM28 [14]. Therefore, mechanistically, the results of this study suggest that RET kinase is a master regulator of hTERT expression by exhibiting control over these kinase signaling cascades, evidenced by the fact that AST-487 inhibits hTERT expression regardless of these other pathways (Figure 7).

In conclusion, this work demonstrates the power of HCS phenotypic drug discovery to identify new therapeutic strategies for repurposing focused clinically relevant compound libraries such as kinase inhibitors. Moreover, the results from this work have significant translational implications when considering that two RET kinase inhibitors, Pralsetinib and Selpercatinib, have recently been FDA-approved for non-small cell lung cancer (NSCLC) and RET+ advanced medullary thyroid cancer. Thus, it would be interesting to measure hTERT expression in patients after receiving Pralsetinib or Selpercatinib therapy. Finally, this work provides potential new precision medicine approaches for cancer patients with up-regulated hTERT expression. This approach may be especially effective in patients harboring mutations/activation of both the RET gene and the hTERT promoter, such as thyroid cancer patients.

## 4. Materials and Methods

### 4.1. Human Cancer Cell Lines

Bladder cancer cell lines UMUC3, UMUC6, UMUC13D, T24T, SW1710, 253J-BV, and UMUC1 were previously described [40]. UMG12, UWG6, UMUC3-CMV-GFP, and UMUC-CMV-hTERT-GFP cell lines are described in Agarwal et al., 2021 [14]. Cell lines were maintained in MEM containing L-glutamine and supplemented with 10% FBS and 1 mM sodium pyruvate. Imaging experiments were performed in phenol red-free medium containing L-glutamine and supplemented with 10% FBS. Cell culturewares and reagents were purchased from Fisher Scientific, USA. All cell lines were confirmed mycoplasma-free (MycoAlert, Lonza, Basel, Switzerland) before screens and cultivated at 37 °C and 5% CO_2_. Cells were utilized for experiments within 4 weeks post-thawing. AST-487 was purchased from Targetmol and SelleckChem.

### 4.2. Monolayer High-Content Screening (HCS)

A manually curated library of 807 kinase inhibitors was created by combining SelleckChem and MedChemExpress kinase inhibitor libraries and removing the duplicate inhibitors. All kinase inhibitors were screened at 3 different concentrations for 1 μM, 100 nM, and 10 nM. Cell plating was performed by the automated Janus Liquid Handler Workstation (PerkinElmer, Waltham, MA, USA), and imaging was done on Opera Phenix High Content Screening System (PerkinElmer). UMG12 cells were seeded at a density of 500 cells/well in 384-well black, clear flat bottom microplates (Greiner Bio-one, Kremsmünster, Upper Austria). Drug vehicle (DMSO at 0.5%) and GFP siRNA (20 nM) were used as a negative and positive control, respectively. Cells were then incubated with kinase inhibitors, with a single drug per well, for 72 h and nuclear stained with Hoechst 33342 dye 20 min before imaging. Cells were imaged with a 20× water objective using 2 channels for Hoechst and GFP. Harmony 4.9 software (PerkinElmer) was employed for image analysis. First, the Hoechst channel was used to find the nuclei, then the cytoplasm and cell numbers were identified based on the GFP channel. The GFP intensity was calculated within cells after background subtraction. Fluorescent images of GFP-positive cells were captured using an EVOS cell imaging system (Thermo Fisher, Waltham, MA, USA) at 20× magnification.

### 4.3. Tumor Organoid High-Content Screening (HCS)

A total of 10,000 cells/well were used for organoid formation. Uniformly suspended cells were seeded in round bottom ultra-low attachment 96-well plates (PerkinElmer). Cells were aggregated by centrifugation at 1000 rpm for 15 min and incubated for 3 days with a 2% growth factor reduced Matrigel (Corning, Corning, NY USA) in phenol red-free RPMI medium 1640. Organoids were treated with the kinase inhibitor library described above at 10 μM concentration. Drug vehicle (0.5% of DMSO) and GFP siRNA (20 nM) were used as a negative and positive control, respectively. All tumor organoids had a diameter above 300 μm and were stained with Hoechst 33342 (Invitrogen, Waltham, MA, USA) 40 min before imaging with the Opera Phenix. The single-tumor organoids were imaged with a 5× air objective (PerkinElmer), recording 15 confocal z-stack images in 20 μm steps and using 3 channels for brightfield, Hoechst, and GFP. Images were analyzed with the Harmony software 4.9 (PerkinElmer). In brief, tumor organoids were analyzed in the maximum projection configuration. Brightfield image was first inverted, then combined with Hoechst and GFP channels. Objects were identified with the combined 3 channels, and the tumor organoid was identified using objective roundness and area as the criteria. The background was defined as the surrounding region of the tumor organoid. The mean GFP intensity of the tumor organoid was calculated after background subtraction.

### 4.4. Hit Validation of Kinase Inhibitors

Hits were validated using BLCA UMG12, UWG6, UMUC3-CMV-GFP, and UMUC3-CMV-hTERT-GFP cells cultured as tumor organoids as described above, and treated with kinase inhibitors at 10 μM concentration for 72 h. High-content imaging and analysis were conducted as described above.

### 4.5. Gene Expression Analysis

Cells in 2D cell culture were scraped after adding lysis buffer (Qiagen RNeasy kit, Hilden, Germany). The subsequent RNA extraction was performed according to the manufacturer’s protocol. For xenograft tumors, total RNA was isolated using the RNeasy Mini Kit (Qiagen). RNA concentration was quantified with the Synergy H1 microplate reader (BioTek, Winooski, VT, USA). cDNA was synthesized using an H-minus Reverse Transcription kit (Fisher Scientific). qPCR reaction was set up with a PowerUP SYBR green master mix (Thermo Fisher). Gene transcript levels were analyzed on the QuantStudio 6 Flex (Applied Biosystems, Waltham, MA, USA) and calculated by the ∆∆CT method for relative quantification using β-Actin as a reference gene. Primer sequences used are provided (Table S1). Xenograft tumor data were analyzed on Graphpad Prism. First, the ROUT method was employed to exclude any outliers (Q = 10%). Data from AST-487 treated group were then normalized to the vehicle-treated group. Normalized data from two separate experiments were combined, and statistical analysis was conducted using an unpaired *t*-test (one-tail). *p*-Values are indicated in Figure 5C,D.

### 4.6. AST-487 Molecular Target Deconvolution Studies

Smartpool genomic siRNAs for all AST-487 gene targets were purchased using cherry-pick library tool of Horizon Discovery. Cells were transfected with 20 nM siRNA in 96-well format using Lipofectamine RNAiMax transfection reagent (Thermo Fisher). After 72 h of transfection, cells were stained with Hoescht 33342 dye and imaged using both Hoescht and GFP channels in the Opera-Phenix imaging system. Post-imaging analysis was done as described above for 2D screening.

### 4.7. AST-487 Dose-Response Studies

BCLS cells (Figure 6) were seeded at 1000 cells/well in 96-well plates. Each condition was done in 6 replicates. Cells were treated with AST-487 at various concentrations and incubated for 72 h before freezing the plate at −80 °C. Frozen plates were then processed using the CyQuant assay (Life Technologies, Carlsbad, CA, USA). Fluorescence intensity was measured using a BioTek microplate reader, and fold change was calculated from differences in intensity of AST-487 compared to DMSO-treated cells.

### 4.8. Tumor Xenograft Studies with AST-487

The in vivo experiments were performed according to IUCAC-approved protocol. For pharmacological inhibition studies, 5–6 weeks aged nude mice were used. The mice were purchased from Taconic biosciences. Two separate animal experiments were carried out with animal number = 5 per group for each experiment. For generating xenograft tumors, 1 million UMG12 and UMUC3-CMV-hTERT-GFP cells were injected subcutaneously to both flanks of each mouse. The mice were ear-tagged, and age-matched male mice were randomized into the vehicle or AST-487 groups. Tumors were measured twice every week, and once tumors reached the appropriate size of ~150 mm^3^, vehicle [1:10 (vol/vol) DMSO-PEG300] and AST-487 (50 mg/kg) were administered by oral gavage. Tumor samples were harvested 6 h post-treatment and snap-frozen in liquid nitrogen. Tumor samples were stored at −80 °C for further analysis. For all in vivo experiments, a blind analysis was performed by investigators among drug treatment groups.

### 4.9. Statistical Analyses

Statistical analyses were performed using Prism v9.0 (GraphPad Software Inc., La Jolla, CA, USA). Data were collected for three independent experiments (N = 3) with three technical replicates (*n* = 3) for each experiment unless otherwise mentioned. For each figure presented, including SI figures, error bars were represented as the SEM from the means of three independent experiments. IC_50_ values were calculated from the best-fit curves within the 95% confidence interval. Statistical significance was calculated using a two-tailed Student’s *t*-test. *p*-value significance is represented as * *p* < 0.05; ** *p* < 0.01; *** *p* < 0.001; **** *p* < 0.0001.

## Data Availability

All data generated or analyzed during this study are included in this published article and its supplementary information files.

## Supplementary Materials

The following supporting information is available at: https://www.mdpi.com/article/10.3390/ijms231810819/s1.

## Sample Availability

Samples of compounds are available from commercial sources.
