# Supplementary material for: AST-487 Inhibits RET Kinase Driven TERT Expression in Bladder Cancer
Source: Int J Mol Sci. 2022 Sep 16;23(18):10819. doi: 10.3390/ijms231810819 (PMC9501578; doi:10.3390/ijms231810819)
Supplement: Supplementary file 1 [file ijms-23-10819-s001.zip › AST-487 (Table S1) 090722.pdf]

Table S1. Primer sequences

Gene name	Direction	Sequence	Assay
hTERT	Forward	CATTTCATCAGCAAGTTTGGAAG	qRT-PCR
	Reverse	TTTCAGGATGGAGTAGCAGAGG	
GFP-hTERT	Forward	CAACGAGAAGCGCGATCA	qRT-PCR
	Reverse	GCGGCATCTTGTACAGCTC	
CDKL3	Forward	CGTTTCCATGGTGGCAAGTG	qRT-PCR
	Reverse	GGGCTTTTACTACGGCGTCA	
HIPK4	Forward	CAGGTCTCGCCTGAGGATGA	qRT-PCR
	Reverse	GGGTCTGGCCTCTCAGCTT	
MAP3K7	Forward	TGGTGCTGAACCATTGCCA	qRT-PCR
	Reverse	TGCAACCAGCAGTAAGTTTGG	
RET	Forward	TCTCAGAGGAGGAGACACCG	qRT-PCR
	Reverse	CGGGTCTGACATGCCATAGA	
β-Actin	Forward	CACTCTTCCAGCCTTCCTTC	qRT-PCR
	Reverse	GTACAGGTCTTTGCGGATGT	
