# Supplementary material for: AST-487 Inhibits RET Kinase Driven TERT Expression in Bladder Cancer
Source: Int J Mol Sci. 2022 Sep 16;23(18):10819. doi: 10.3390/ijms231810819 (PMC9501578; doi:10.3390/ijms231810819)
Supplement: Supplementary file 1 [file ijms-23-10819-s001.zip › AST-487 (Table S2) 090722.pdf]

Table S2. List of kinase targets of potential hits identified from HCS.

		Number of Kinase inhibitor hits		
	Known to regulate hTERT from Literature	3D screen	2D screen	Hits overlapped in 3D & 2D screens
**PI3K**	Yes	38	12	11
**CDK**	No	27	3	2
**EGFR**	Yes	26	16	11
**mTOR**	Yes	28	15	13
**Akt**	Yes	16	7	7
**Raf**	Yes	12	5	4
**c-Met**	No	10	9	4
**JAK**	No	11	3	1
**MEK**	Yes	10	10	3
**VEGFR**	No	10	4	3
**ALK**	No	8	2	1
**ATM/ATR**	No	7	2	1
**Bcr-Abl**	Yes	6	4	2
**DNA-PK**	No	6	0	0
**p38 MAPK**	Yes	6	16	5
**FGFR**	No	5	2	1
**ERK**	Yes	4	4	1
**FLT3, JAK, PKC**	No	4	3	1
**c-RET,VEGFR**	No	3	1	1
**PDGFR, c-Kit, VEGFR**	No	3	2	1
**CSF-1R, Raf**	No	2	1	1
**HER2**	No	2	1	1
**IRAK**	No	2	1	1
**PDK-1**	No	2	1	1
**HIPK4**	No	1	1	1
