# Supplementary material for: AST-487 Inhibits RET Kinase Driven TERT Expression in Bladder Cancer
Source: Int J Mol Sci. 2022 Sep 16;23(18):10819. doi: 10.3390/ijms231810819 (PMC9501578; doi:10.3390/ijms231810819)
Supplement: Supplementary file 1 [file ijms-23-10819-s001.zip › Supplemental Information.pdf]

SUPPLEMENTAL INFORMATION

Figure S1. AST-487 suppresses the promoter driven expression of GFP-hTERT. Representative images of UMG12 and UWG6 cells showing changes in intensity of GFP-hTERT after treatment with AST-487 at indicated concentrations for 72h. DMSO was used as control, scale bar is 150μM.

Figure S2. Pan-cancer expression of hTERT in normal and tumor tissues taken from TCGA database.

Figure S3. Analysis of co-occurrence of RET and TERT alterations using cBioPortal web site (https://www.cbioportal.org/) and tumor data from reference [10].

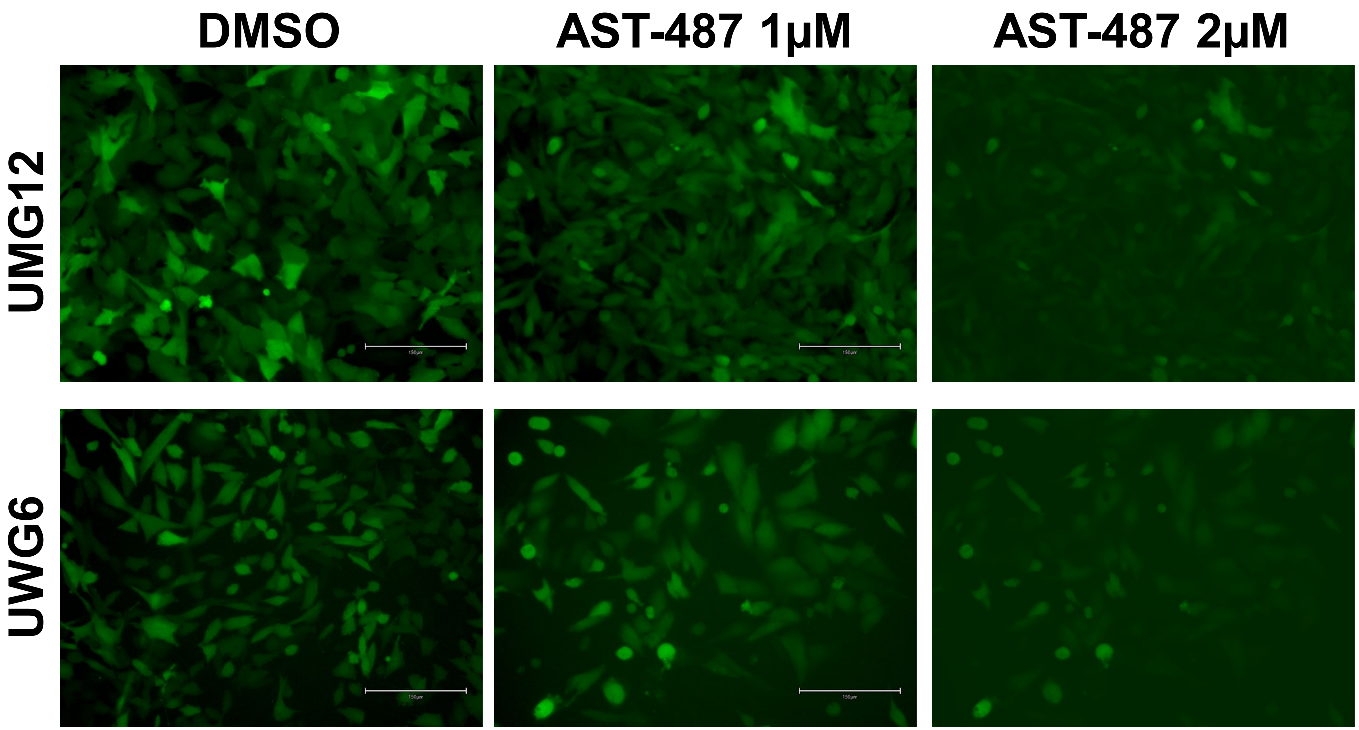

Figure S1

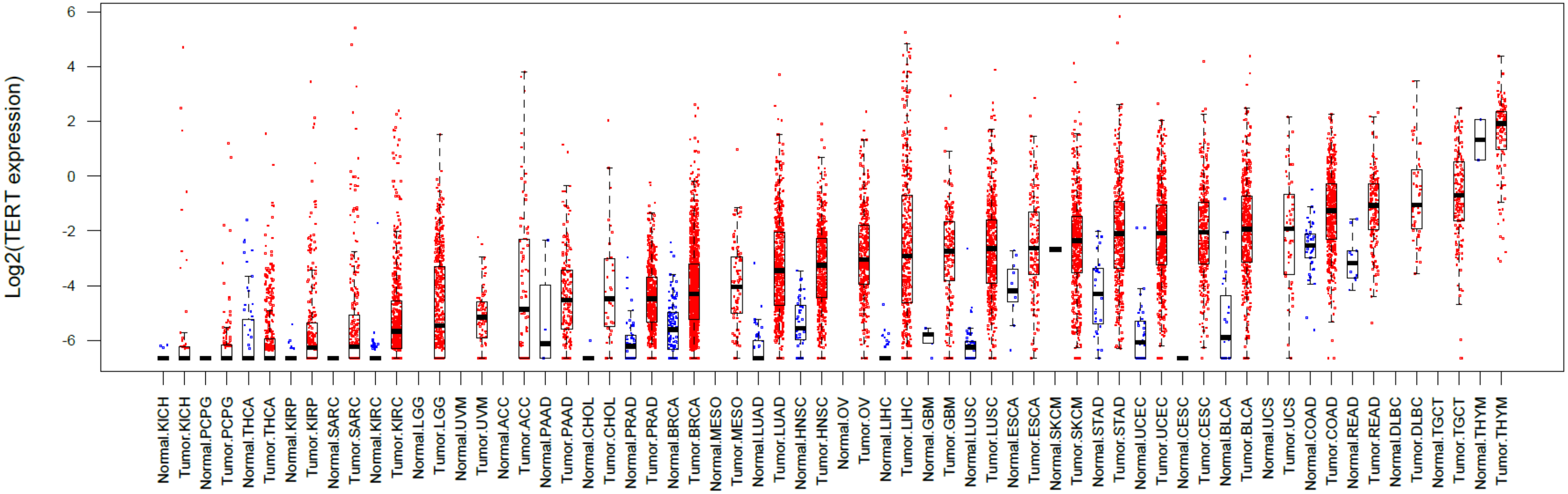
Log2(TERT expression)
6
4
2
0
-2
-4
-6
Normal.KICH
Tumor.KICH
Normal.PCPG
Tumor.PCPG
Normal.THCA
Tumor.THCA
Normal.KIRP
Tumor.KIRP
Normal.SARC
Tumor.SARC
Normal.KIRC
Tumor.KIRC
Normal.LGG
Tumor.LGG
Normal.UVM
Tumor.UVM
Normal.ACC
Tumor.ACC
Normal.PAAD
Tumor.PAAD
Normal.CHOL
Tumor.CHOL
Normal.PRAD
Tumor.PRAD
Normal.BRCA
Tumor.BRCA
Normal.MESO
Tumor.MESO
Normal.LUAD
Tumor.LUAD
Normal.HNSC
Tumor.HNSC
Normal.OV
Tumor.OV
Normal.LIHC
Tumor.LIHC
Normal.GBM
Tumor.GBM
Normal.LUSC
Tumor.LUSC
Normal.ESCA
Tumor.ESCA
Normal.SKCM
Tumor.SKCM
Normal.STAD
Tumor.STAD
Normal.UCEC
Tumor.UCEC
Normal.CESC
Tumor.CESC
Normal.BLCA
Tumor.BLCA
Normal.UCS
Tumor.UCS
Normal.COAD
Tumor.COAD
Normal.READ
Tumor.READ
Normal.DLBC
Tumor.DLBC
Normal.TGCT
Tumor.TGCT
Normal.THYM
Tumor.THYM

Figure S2

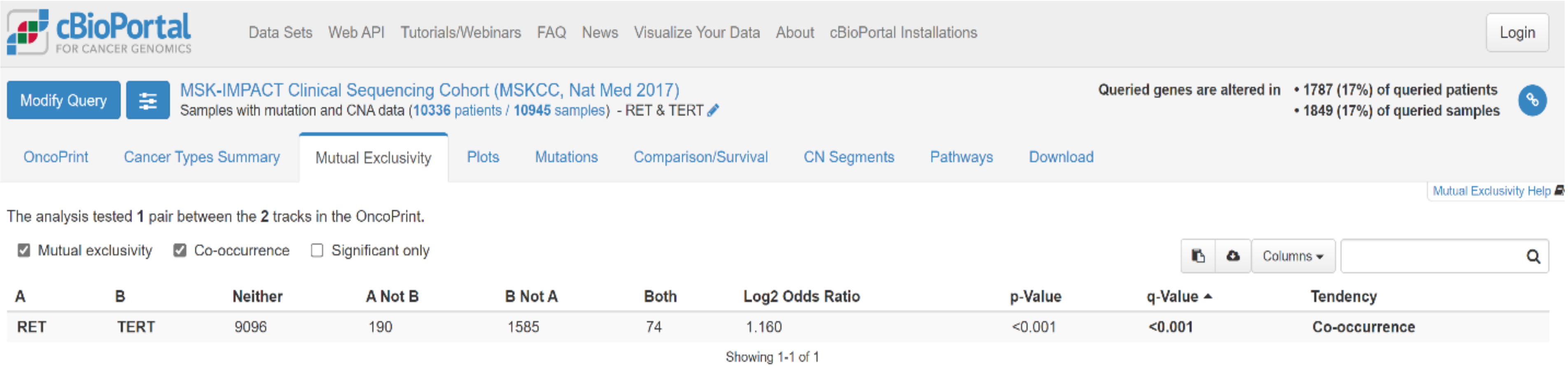

A	B	Neither	A Not B	B Not A	Both	Log2 Odds Ratio	p-Value	q-Value	Tendency
RET	TERT	9096	190	1585	74	1.160	<0.001	**<0.001**	**Co-occurrence**

Figure S3
